# Copper Rich Composite Materials Based on Carboxylic Cation Exchangers and Their Thermal Transformation

**DOI:** 10.3390/polym13183199

**Published:** 2021-09-21

**Authors:** Elżbieta Kociołek-Balawejder, Ewa Stanisławska, Irena Jacukowicz-Sobala, Igor Mucha

**Affiliations:** 1 Department of Industrial Chemistry, Wroclaw University of Economics and Business, 53-345 Wrocław, Poland; ewa.stanislawska@ue.wroc.pl (E.S.); irena.jacukowicz@ue.wroc.pl (I.J.-S.); 2 Department of Analytical Chemistry, Wroclaw Medical University, 50-556 Wrocław, Poland; igor.mucha@umed.wroc.pl

**Keywords:** carboxylic cation exchanger, cuprous oxide, metallic copper, composite, thermal analysis, incineration, pyrolysis

## Abstract

The effect of a cupric deposit (Cu^2+^, CuO) on the thermal decomposition of carboxylic cation exchangers (CCEs) is not known, and such studies may have practical significance. CCEs have a very high ion exchange capacity, so an exceptionally large amount of CuO (which is a catalyst) can be precipitated inside them. Two CCEs, macroreticular (Amberlite IRC50) and gel-like (Amberlite IRC86), served as a polymeric support to obtain copper-rich hybrid ion exchangers. Composites with CuO particles inside a polyacrylic matrix (up to 35.0 wt% Cu) were obtained. Thermal analyses under air and under N_2_ were performed for CCEs in the H^+^ and Cu^2+^ form with and without a CuO deposit. The results of sixteen experiments are discussed based on the TG/DTG curves and XRD patterns of the solid residues. Under air, the cupric deposit shifted the particular transformations and the ultimate polymeric matter decomposition (combustion) toward lower temperatures (even about 100–150 °C). Under N_2_, the reduction of the cupric deposit to metallic copper took place. Unique composite materials enriched in carbonaceous matter were obtained, as the products of polymeric matrix decomposition (free radicals and hydrogen) created an additional amount of carbon char due to the utilization of a certain amount of hydrogen to reduce Cu (II) to Cu^0^.

## 1. Introduction

Activated carbon, biochar, zeolite, synthetic polymers, and biopolymers supporting metal oxide nanoparticles are modern and effective composite materials that offer properties and application opportunities not exhibited separately by the individual components–high molecular weight host materials or inorganic nanoparticles alone [1,2,3,4,5,6]. The composite materials display more advantageous properties than raw supporting materials, allowing the effective removal of troublesome organic contaminants, such as antibiotics, dyes, and pesticides, and also toxic metallic and nonmetallic substances from aqueous media [7,8,9,10]. 

By dispersing metal oxide nanoparticles into an ion exchanger matrix, hybrid ion exchangers (HIXs) can be prepared. Due to their form of porous spherical beads with mechanical strength and due to overcoming the propensity of parent nanoparticles to aggregate (by isolating them within the polymeric skeleton), HIXs are suitable for batch and flow-through systems, providing a highly accessible surface area, high sorption capacity, fast kinetics, and enhanced selectivity in purification processes (the Donnan membrane effect controls what type of ions can enter inside the resin structure) [11]. The different morphology of the host materials (ion exchanger macroreticular vs. gel-like structure), the diverse nature and concentration of the ionogenic functional groups (strongly or weakly acidic vs. strongly or weakly basic), the various methods of dispersing metal oxide NPs inside the polymer support, and the specific properties of immobilized metal oxide nanoparticles all contribute to the diversity of their applications, including separation processes, catalysis and biocidal actions [12,13,14,15,16,17]. The most popular HIXs are FeOOH, MnO_2_, and ZrO_2_ doped anion exchangers. They are used to remove arsenic species and various harmful oxyanions from water through sorption and redox processes [18,19,20].

The introduction of metal oxides into the ion exchanger matrix can have various consequences, including catalysis related. In 2002, a study demonstrated that metal compounds including CuSO_4_·5H_2_O, CuO, and FeSO_4_·7H_2_O (mixed with ionite), as well as Cu^2+^ ions pre-loaded on the resin, had a catalytic effect on the oxidative pyrolysis of cationic (sulfonic) resin, in which the decomposition of functional groups and polymer matrices was enhanced [21]. Thermal decomposition of ion exchangers by combustion or pyrolysis is one of the possibilities for the treatment and disposal of spent ion exchangers, reducing their volume [22,23,24,25], making the waste more stable [26,27] and giving usable porous carbon beads [28,29,30,31,32]. Handling spent ion exchangers is especially environmentally significant if they come from nuclear power plants and constitute a highly radioactive waste [33,34,35,36]. Considering the catalytic properties of copper compounds and also the necessity to develop methods of treatment of the used ion exchangers, we have undertaken research on the synthesis and thermal decomposition of HIXs under air and under N_2_, in which, as host materials for CuO, Cu (OH)_2_, Cu_4_(OH)_6_SO_4_, Cu_2_O, and Cu^0^ fine particles, strongly basic anion exchangers with macroreticular and gel-like structure were used [37,38,39]. It turned out that copper compounds deposited in the skeleton of such resins significantly altered the results of the thermal analysis in comparison with those of pure resin [40,41]. The differences resulted from the process end temperature, the acceleration of the decomposition steps, the amount of carbonizate in the post-pyrolysis residue, and also the composition of the inorganic phase (CuO after combustion, Cu^0^ after pyrolysis). It was demonstrated that during the thermal decomposition of the HIXs under N_2_, the oxidation state of the copper atom in the deposit had an increasing effect (cupric compounds > cuprous compounds > metallic copper) on the amount of the forming char. Owing to the hydrogen-consuming conversion (reduction of copper compounds occurred), much more carbonizate formed than in the pyrolysis of pure resin. It has been shown that it is possible to use thermal processes for transforming spent HIXs into carbonaceous materials containing a substantial amount of metallic copper-potential reagents for purification and catalytic processes [42,43,44].

Considering the beneficial effect of CuO particles on the speed of thermal decomposition and the mass of solid residue formed during HIX pyrolysis, we have expanded research on this issue by synthesizing materials that may contain a much larger amount of this oxide than before, which can also give residual carbonaceous materials extremely rich in metallic copper. As polymeric hosts, carboxylic cation exchangers (CCEs) with –COOH groups having both macroreticular and gel-like structure were used. As a result of the low molecular weight of the aliphatic carboxylic acid (acrylic acid or methacrylic acid) forming the polymeric skeleton, their ion exchange capacity (the number of ionogenic groups per mass unit) is several times higher than the sorption capacity of anion exchangers with trimethyl ammonium functional groups used previously as support for CuO [45,46,47]. As regards the synthesis of cationites with a CuO deposit, this has so far only been performed for strongly acidic sulfonic cationite receiving HIX for low-concentration ammonia nitrogen removal from water [48]. A CCE with a CuO deposit has not yet been described, but the stabilization of metallic copper nanoparticles inside the CCE matrix has been reported [49,50]. 

CCEs are weakly acidic resins, highly selective with respect to the H^+^ ion and in contrast with strongly acidic ones, first of all sorb hydrogen ions from among all cations (in this form, they do not split neutral salts). A small, stoichiometric excess of a strong mineral acid is sufficient to convert the resin into the H^+^ form. The degree of ionization of their less-dissociated active groups corresponds to that of acetic acid. The effective pH range is between 6 and 14. A relatively high degree of affinity exists for the earth alkaline metal ions. CCEs are used primarily for the removal of cations from basic solutions (dealkalization, softening of high salinity waters), for the splitting of weakly alkaline salts of polyvalent cations, and also for the isolation and purification of biochemicals such as antibiotics, vitamins, alkaloids, and enzymes [51,52,53,54,55]. The use of CCEs for reducing the volume of radioactive waste generated in nuclear power plants was also considered [56,57].

Given the high sorption capacity of CCEs (which is many times higher than other ion exchange resins), the aim of this work was to synthesize HIXs rich in CuO fine particles and examine their thermal properties compared to pure resins. The research was intended to answer the following questions. How much CuO can be introduced into the skeleton of both cationites, and does the gel-type structure limit the mass of this deposit? Can CCEs containing CuO be used in the H^+^ form without losing the deposit? What is the effect of the ionic form of the functional groups (Cu^2+^, H^+^) and CuO deposit on the decomposition of both resins under air and N_2_ atmospheres? During pyrolysis, will the cracked polyacrylic matrix containing –COOH groups (with oxygen atoms) reduce Cu (II) and create an additional amount of carbon char (as in the case of polystyrene matrix of anion exchange resins)? What is the mass and composition of the solid residue depending on the thermal analysis atmosphere?

The research described here concerns the synthesis of HIXs particularly rich in a cupric deposit, shows the effects of various factors on the thermal decomposition of synthesized composite materials, and is also related to an important area of modern research–carbothermal synthesis of metal–char composites, which are forward-looking materials. Thanks to the presence of dispersed metallic copper fine particles within the carbonaceous phase, they represent reagents that can be useful for mediating environmentally important processes. 

## 2. Materials and Methods

### 2.1. Materials

The polymeric supports for CuO were Amberlite IRC50 (macroporous) and Amberlite IRC86 (gel-like), commercial weakly acidic cation exchangers produced by The Dow Chemical Co. (Table 1). All the chemicals used in this study, including CuSO_4_·5H_2_O, CH_3_COOH 80%, an analytically weighed amount of ethylenediaminetetraacetic acid disodium salt dihydrate (EDTA) 0.05 M (Chempur, Piekary Śląskie, Poland), murexide (Eurochem, Tarnów, Poland), HCl 35–38%, ammonia solution 25%, and NaOH (PPH Stanlab, Lublin, Poland), were of analytical grade. All the solutions were prepared using deionized water.

### 2.2. Synthesis of HIXs 

**Scheme 1:**
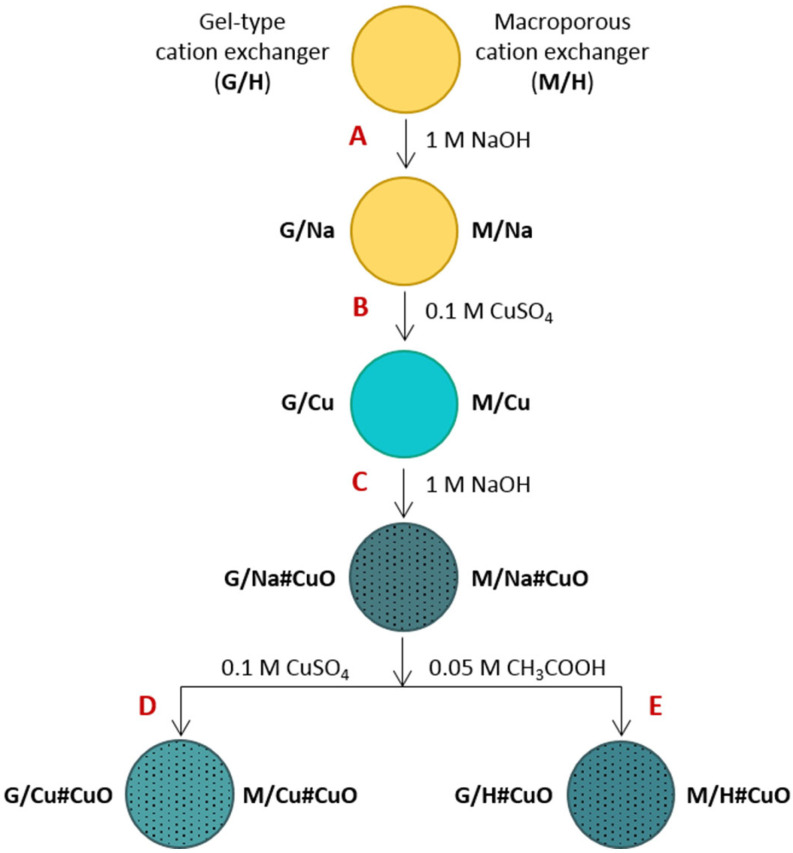
Synthesis of HIXs containing CuO deposit within the matrix of carboxylic cation exchangers (A, B, D, E–ion exchange reactions, C–precipitation of CuO in alkaline medium).

Before chemical transformation, the M/H and G/H samples (i.e., the functional groups of cationites in the H^+^ form) were soaked and washed with distilled water and dried in a dryer at 40 °C for 24 h. Both resins were transformed in five stages. Four of the transformations (A, B, D, and E) consisted of ion exchange, quantitatively carried out on the functional groups of the cationite in the Na^+^, Cu^2+^ and back in the H^+^ form using solutions such as 1 M NaOH, 0.1 M CuSO_4_, and 0.05 M CH_3_COOH. In each case, the process was conducted in a column until the concentrations of effluent and influent were approximately the same. Stage C, consisting of precipitating CuO in the resin matrix, was conducted batchwise, shaking for 4 h M/Cu or G/Cu with excess of 1 M NaOH at ambient temperature. The sequence of transformations is illustrated in Scheme 1.

The Cu content in the samples was determined after dissolving the cupric deposit in 2 M HCl (0.5 g sample, 20 cm^3^ solution, 30 min of shaking). The solution was alkalized with concentrated ammonia solution and titrated with 0.05 M EDTA against murexide. 

### 2.3. Thermogravimetric Analysis

The thermogravimetric experiment was carried out using a TG 209 F1 Libra thermogravimetric analyzer (Netzsch, Selb, Germany). For the thermogravimetric analysis (TG/DTG), the samples were placed in an alumina crucible (150 μL). About 14 mg of sample was heated from an ambient temperature up to 950 °C at a heating rate of 10 °C/min. Experiments were performed in a nitrogen atmosphere (30 mL/min) and in an oxidative atmosphere (protective gas—nitrogen 10 mL/min, and purge gas—air 30 mL/min). The measurements were performed under exactly the same conditions and repeated to determine their reproducibility, which was found to be very good. The sample curves were analyzed using Netzsch Proteus 7.1.0 software (Selb, Germany) The temperatures and mass changes were given with an accuracy (standard deviation) of ±1.5 K and 0.4%, respectively.

### 2.4. X-ray Powder Diffraction Analysis

The Bruker D2 Phaser X-ray diffractometer (Bruker AXS, Karlsruhe, Germany) operating in Bragg–Brentano (θ/2θ) geometry, equipped with a LYNXEYE detector (based on silicon strip technology) and copper anode (Cu-Kα), was used to collect the data for this study. The powdered samples were analyzed on a zero-background sample holder. All measurements were taken at room temperature using a step scan mode with the step of 0.02° (2θ) and 0.2 s per step across the angular range 5° ≤ (2θ) *≤* 70°. The optics of the D2 PHASER diffractometer comprised a system of a Soller slit module with 2.5°, a divergence slit with 0.6 mm and a nickel filter. The X-ray tube operated at 30 kV and 10 mA. The collected XRD patterns were evaluated using Diffrac.EVA v.3.2 (Bruker AXS, Karlsruhe, Germany) software.

## 3. Results and Discussion

### 3.1. Hybrid Polymer Formation 

To obtain novel HIXs with high CuO content, we used two carboxyl cation exchangers (Table 1), which show the highest ion exchange capacity (almost 11.0 meq/g) among mass-produced ion exchangers. These resins contained the same functional groups and in a comparable amount, but they differed in the structure and chemical composition of the polymeric skeleton. The matrix of the macroreticular cation exchanger was obtained by polymerizing a methacrylic acid with divinylbenzene (DVB) as a cross-linking agent, whereas the matrix of the gel-like cation exchanger was obtained by polymerizing an acrylic acid with DVB (the precise producer data concerning the cross-linking degree value are not known). The acrylic resins are slightly more strongly acidic (more dissociated, pK_a_ 4.8) than the methacrylic acid products (less dissociated, pK_a_ 5.7) [58]. 

Reaction equations illustrate the transformations to which both cation exchangers were subjected to dope CuO Equations (1)–(5):

(1) $$\left[P\right]-\mathrm{COOH}+\mathrm{NaOH}\;\to \left[P\right]-\mathrm{COONa}+H_{2}O\;$$

(2) $$2\left[P\right]-\mathrm{COONa}+{\mathrm{CuSO}}_{4}\;\to {\left(\left[P\right]-\mathrm{COO}\right)}_{2}\mathrm{Cu}+{\mathrm{Na}}_{2}{\mathrm{SO}}_{4}$$

(3) $${\left(\left[P\right]-\mathrm{COO}\right)}_{2}\mathrm{Cu}\;\overset{\text{excess NaOH}}{\to }\;\{2\left[P\right]-\mathrm{COONa}\}\#\mathrm{CuO}$$

(4) $$\{2\left[P\right]-\mathrm{COONa}\}\#\mathrm{CuO}+{\mathrm{CuSO}}_{4}\;\to {\left(\left[P\right]-\mathrm{COO}\right)}_{2}\mathrm{Cu}\#\mathrm{CuO}+{\mathrm{Na}}_{2}{\mathrm{SO}}_{4}$$

(5) $$\left[P\right]-\mathrm{COONa}\#\mathrm{CuO}+{\mathrm{CH}}_{3}\mathrm{COOH}\;\to \left[P\right]-\mathrm{COOH}\#\mathrm{CuO}+{\mathrm{CH}}_{3}\mathrm{COONa}$$

[P]—polymeric sceleton of cation exchanger.

Three types of materials were synthesized: with the functional groups in the Na^+^ form (M/Na#CuO, G/Na#CuO), with the functional groups in the Cu^2+^ form (M/Cu#CuO, G/Cu#CuO), and with the functional groups in the H^+^ form (M/H#CuO, G/H#CuO). As the functional groups of both host materials occurred in the H^+^ form (M/H, G/H), consequently, they cannot remove Cu^2+^ ions from solutions of mineral acid salts (because the acid produced lowers the pH and prevents further ion exchange); firstly, they had to be converted into the Na^+^ form. For this purpose, 1 M NaOH was used, initially in the batch method and then in the column, as the reaction (Equation (1)) was running with a thermal effect and with a large increase in the volume of the bed (H^+^ *→* Na^+^ swelling-in-water 80–100%). The functional groups in the Na^+^ form could already be converted into the Cu^2+^ form by passing 0.1 M CuSO_4_ solution through the resin bed in column (Equation (2)). Then, the resin was shaken with an excess of NaOH solution in batch condition to precipitate CuO in its matrix (Equation (3)). To obtain HIX with a simpler elementary composition, the functional groups were converted into the Cu^2+^ form (Equation (4)). The aim of transformation of M/Na#CuO and G/Na#CuO to M/H#CuO and G/H#CuO (Equation (5)) was to obtain HIXs that would not alkalize the reaction environment, as high pH makes many sorption and catalytic processes inefficient. To perform such gentle transformation effectively, i.e., to prevent the dissolution of the deposit in acidic medium, 0.05 M CH_3_COOH (pH ~ 3.9) was used, considering that the degree of ionization of the –COOH groups in resins corresponds to that of acetic acid. Therefore, CH_3_COOH solution was passed through the M/Na#CuO or G/Na#CuO bed in the column until traces of Cu^2+^ appeared in the effluent. In the case of M/Na#CuO, this was done at pH ~ 6.0, and in the case of G/Na#CuO at pH ~ 4.7. This result confirmed the association of the acidity of functional groups with the chemical composition of the resin (polymethacrylic acid vs. polyacrylic acid) [58]. At the beginning of the column process, the pH of the effluent was ~10.5, due to the presence of CH_3_COONa.

**Figure 1:**
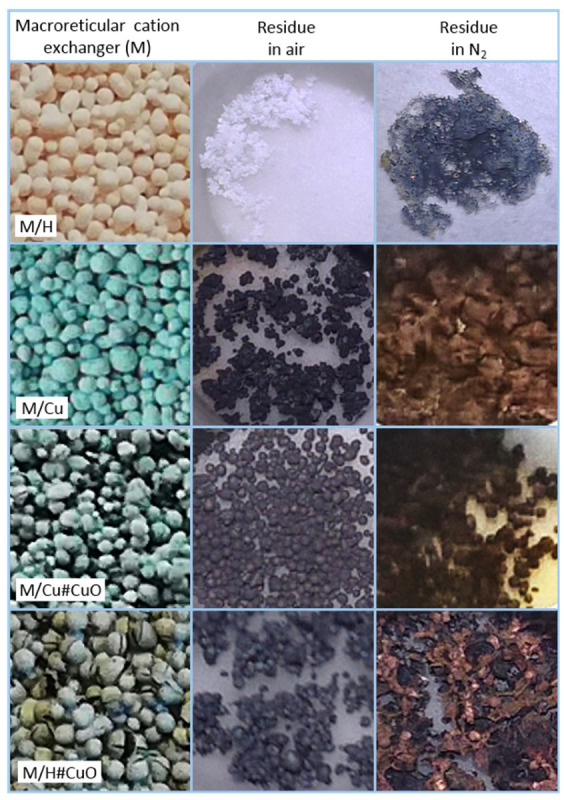
Photographs of examined materials based on macroporous carboxylic cation exchanger (left column), solid residues after their thermal analysis in air (middle column) and in N_2_ (right column).

**Figure 2:**
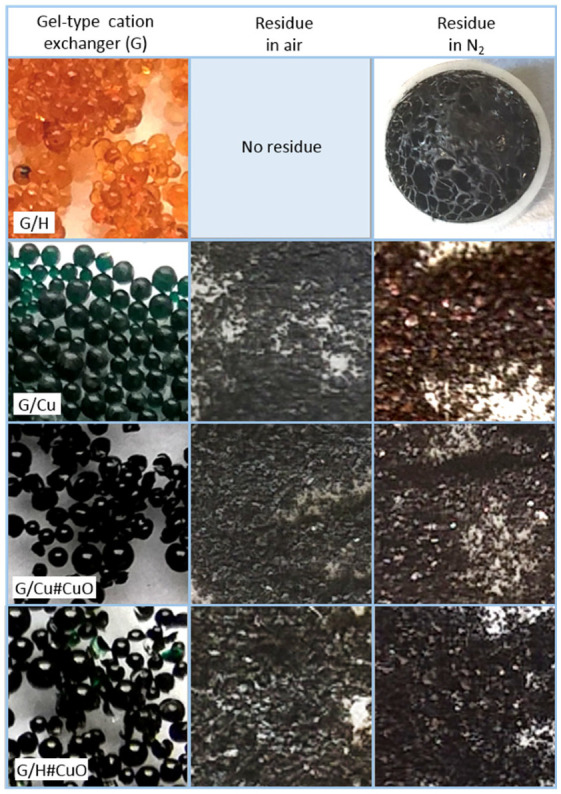
Photographs of examined materials based on gel-type carboxylic cation exchanger (left column), solid residues after their thermal analysis in air (middle column) and in N_2_ (right column).

Table 2 collects data characterizing reaction products to better interpret their composition. Even though “Cu content in sample” (penultimate column in Table 2) expresses the actual copper content in this sample (per mass unit), these results did not fully reflect the degree of transformation. Practically for each reaction/transformation, the mass of the product differed significantly from the mass of the substrate, not only as a result of chemical reactions but also as a result of different hydration of functional groups depending on their ionic form. Considering the gain/decrease in sample mass, one can estimate that all transformations were carried out virtually quantitatively, and the obtained products contained up to 35% wt% Cu. Figure 1 and Figure 2 (first columns) show photographs of two starting resins and products derived from them. 

### 3.2. Thermal Analysis in Air

In order to achieve the second objective of the work, to assess the effect of copper (II) on the thermal decomposition of examined resins and to identify the solid decomposition products, materials with a homogeneous elementary composition, containing copper atoms (not sodium), were selected for analysis. Eight materials—two starting cation exchangers (M/H, G/H), two cation exchangers in the Cu^2+^ form (M/Cu, G/Cu), two cation exchanger in the Cu^2+^ form with a CuO deposit (M/Cu#CuO, G/Cu#CuO), and two cation exchanger in the H^+^ form with a CuO deposit (M/H#CuO, G/H#CuO)—were subjected to thermal analysis. The results are grouped according to the structure of the polymeric support (macroreticular or gel-like) and the medium in which the process proceeded (air or N_2_). TG/DTG curves of sixteen experiments with numerical data characterizing the particular transformation, their temperature range, and the temperature at which the maximum decomposition rate, end temperature, mass change, and residual mass occurred are presented. These findings give an opportunity to analyze such phenomena as the effect of the ionic form of the carboxylic groups on the water content of the material.

**Scheme 2:**
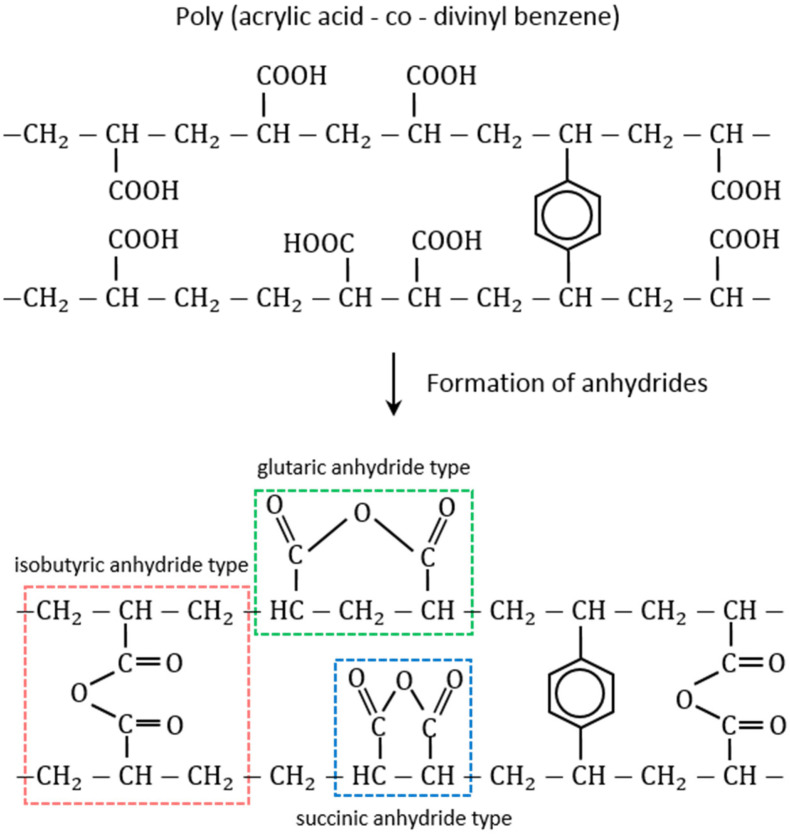
Formation of anhydrides during thermal decomposition of carboxylic cation exchangers.

**Figure 3:**
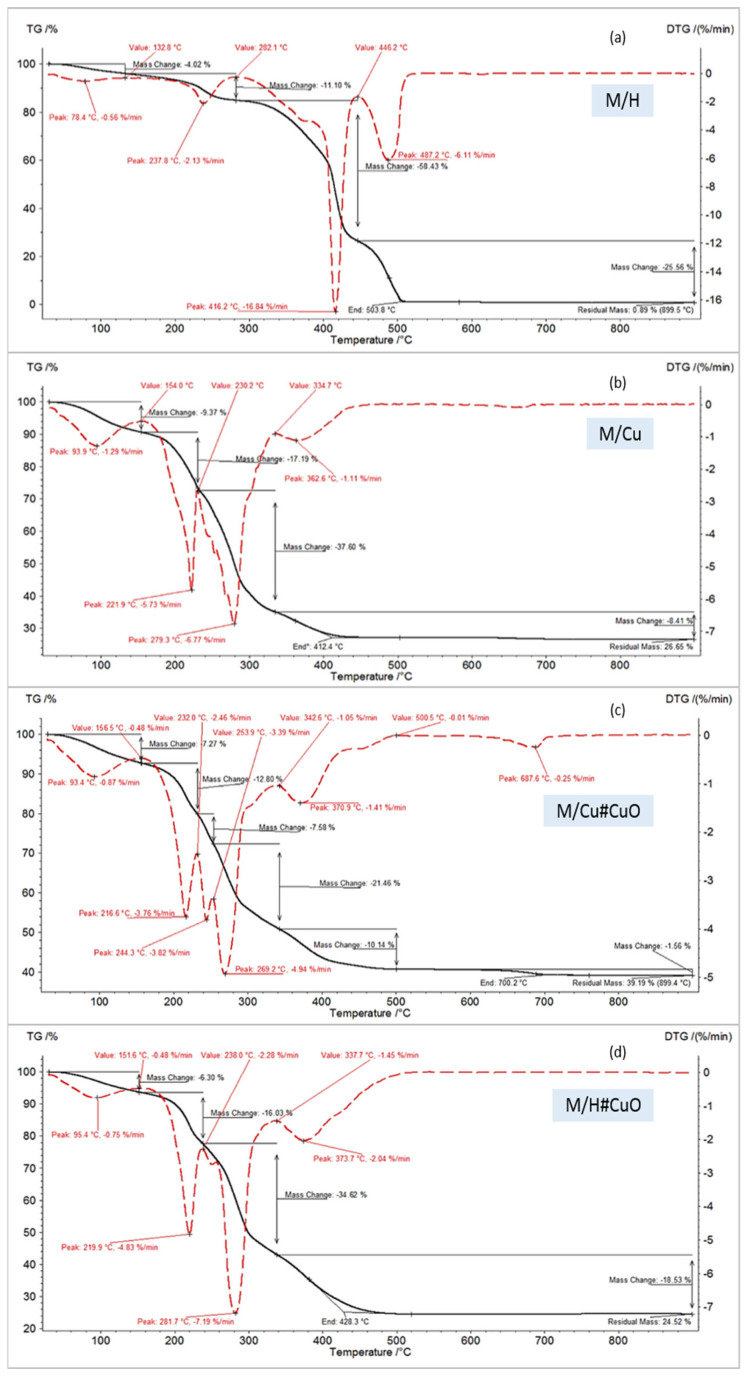
TG/DTG curves in air of (**a**) M/H, (**b**) M/Cu, (**c**) M/Cu#CuO, and (**d**) M/H#CuO.

Figure 3a shows the TG/DTG curves recorded in air atmosphere for M/H, in which the existence of four decomposition steps associated with mass losses can be observed. The first decomposition step up to 132.8 °C (maximum decomposition rate at 78.4 °C) with a mass loss of 4.0% corresponds to the elimination of osmotic water molecules existing in the pores of resin and hydrogen-bonded water (from hydration sites of carboxylic groups). Compared to the ion exchangers of various type [40,41,59,60], this result is very low and indicates that CCE in the H^+^ form is especially weakly hydrated. The second step up to 282.1 °C (maximum decomposition rate at 237.8 °C) with a mass loss of 11.1% can be assigned to the water elimination between two neighboring carboxylic groups with the creation of some polyacrylic cyclic anhydrides by intermolecular dehydration (5-atom ring such as succinic anhydride type or 6-atom ring such as glutaric anhydride type, Scheme 2). The third step up to 446.2 °C (maximum decomposition rate as much as 16.8%/min at 416.2 °C) with a mass loss of 58.4% is the most intense transformation in this experiment, and this is a decarboxylation process of the previously dehydrated structure (polyanhydride decomposition through decarboxylation and elimination of CO_2_ and CO) [61,62,63,64,65]. The last decomposition step up to 503.8 °C corresponded to the oxidative degradation (combustion) of polymeric matrix when oxygen (~20.8% volume) interacts with the hydrocarbon chain and its degradation products. The residue left at 900 °C is ash and is about 0.9% of the original sample. The described transformations due to the accompanying changes and mass losses corresponded well to the results described in the work [62], where by mass spectrometry, volatile process products were identified, and FTIR analysis confirmed the formation of polymeric anhydrides as intermediate products of CCE thermal decomposition.

The TG/DTG curves of M/Cu, M/Cu#CuO, and M/H#CuO in air (Figure 3b–d) have a similar course but are significantly different in comparison to the parent resin (Figure 3a). When analyzing the data from these three experiments, it should be taken into account that although organic matter in them dominates, it represents no more than 75% of their mass (and in one case only 60%). For this reason, the mass loss for the particular stages is smaller than in the case of the parent resin. Analyzing the first transformation corresponding to the evaporation of water molecules, one can see that the samples in which the functional groups in the Cu^2+^ form are much more hydrated (a greater mass loss is observed on the TG curve) than the samples containing the functional groups in the H^+^ form. In each experiment, the presence of copper in the tested material, as Cu^2+^ ions in functional groups and as CuO particles in the resin matrix, significantly accelerated the thermal decomposition of the host material. The decomposition of these samples ended at a temperature 100 °C lower than that of M/H alone. The significant acceleration of thermal decomposition can be observed in a temperature range of 200–300 °C, where there are two broad peaks indicating intense decomposition of functional groups, and especially very fast decarboxylation (at a temperature of about 150 °C lower than pure resin). All residues left at about 900 °C had a mass slightly higher than the copper content in the output sample. In the case of the sample containing the most copper (M/Cu#CuO, 32.7 wt% Cu), the residue represented about 39.2 wt% of the original sample.

**Figure 4:**
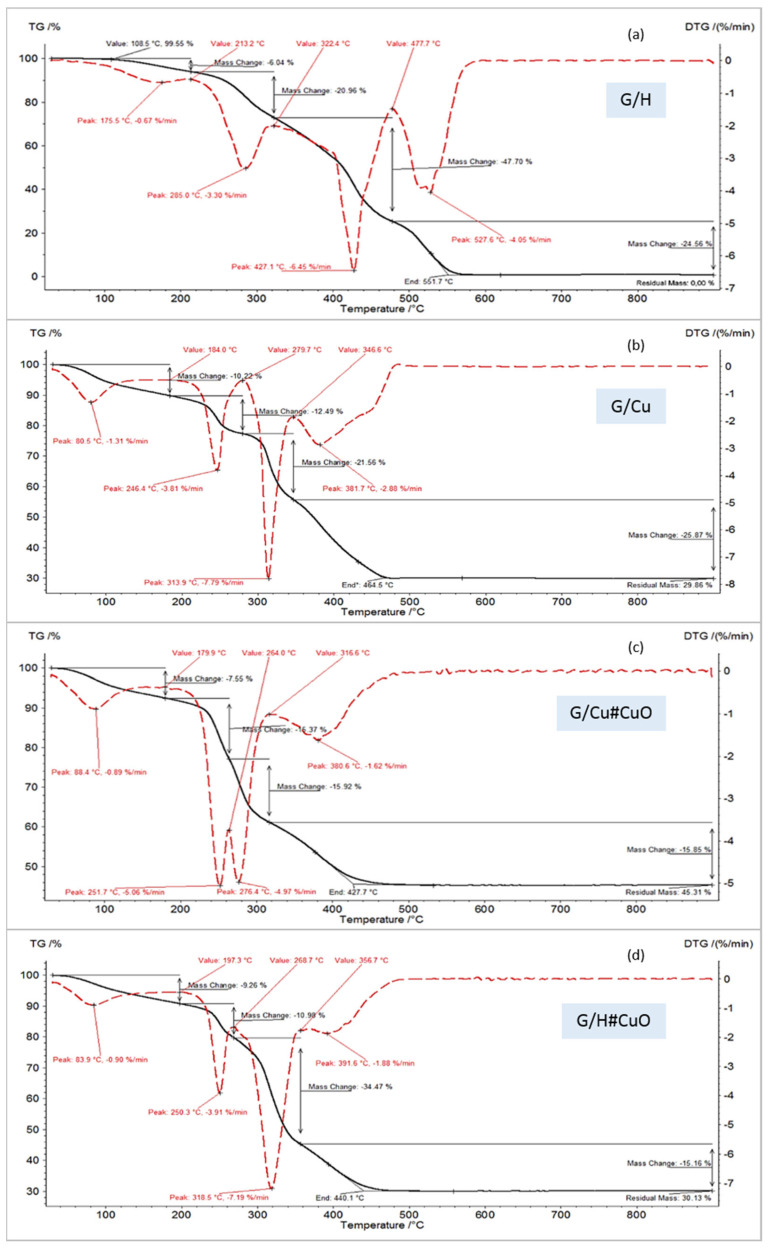
TG/DTG curves in air of (**a**) G/H, (**b**) G/Cu, (**c**) G/Cu#CuO, and (**d**) G/H#CuO.

Figure 4 shows the results of similar studies in air, in which as support for copper(II), the gel-like cation exchanger was used. According to Figure 4a, dehydration of G/H (maximum decomposition rate at 175.5 °C with a mass loss of 6.0%) was proceeding at a higher temperature than dehydration of M/H (maximum decomposition rate at 78.4 °C, Figure 3a). Again, it turned out that the resin in the H^+^ form contained little water, and the compact structure of the gel matrix without real pores (strongly shrinking during drying) made it difficult for water molecules to escape outside [39]. A significant difference in the thermal decomposition of G/H compared to M/H was observed at 200–300 °C. A mass loss of as much as 21.0% (maximum decomposition rate at 285.0 °C) indicated that for G/H, the changes taking place went beyond water elimination between two neighboring carboxylic groups with the creation of some polyacrylic cyclic anhydrides by intramolecular dehydration, as in the case of M/H. The intermolecular dehydration probability with the isobutyric anhydride formation (Scheme 2) resulted from the compact structure of the dry gel matrix and the proximity of polymer chains [62,64,65].

The second columns in Figure 1 and in Figure 2 show the residues after thermal decomposition in air. Residues in Figure 2 do not have the shape of beads, as is the case in Figure 1. Studies on the thermal decomposition of three copper-containing samples obtained with gel-like resin gave an unexpected result: during heating, the balls “clicked” and “jumped” out of the crucible, making the resulting TG/DTG curves uninterpretable. Three samples were therefore thermally analyzed after grinding in an agate mortar (Figure 4b–d). Again, the thermal decomposition of materials containing Cu (II) occurred much more easily (at a lower temperature) than the thermal decomposition of the host material (end temperature respectively 464.5, 427.7, and 440.1 °C vs. 551.7 °C). For the gel-like host material, the copper contained in it accelerated the decarboxylation step by almost 150 °C (G/Cu#CuO maximum decomposition rate at 276.4 °C, vs. G/H at 427.1 °C). 

**Figure 5:**
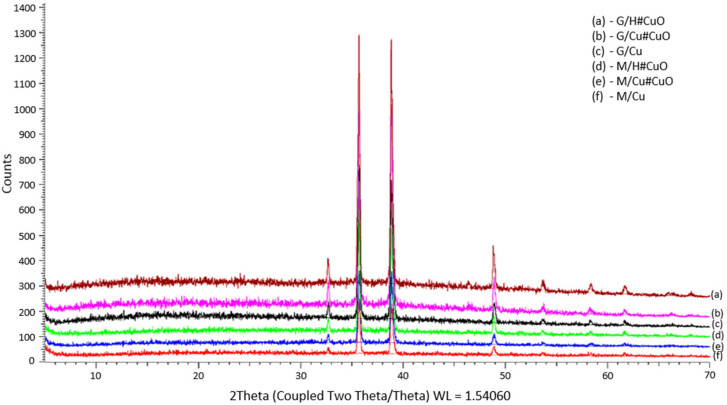
XRD patterns of residues in air.

The results of thermal decomposition of eight samples in air are presented in Table 3. From data showing the peak temperatures and end temperature of polymer degradation, it can be noted that, in any case, the presence of copper in the sample (as Cu^2+^ ion or CuO deposit) shifted the polymeric matter decomposition toward lower temperatures. Due to the high copper content of the test samples, the mass of the combustion residues was also high. An XRD analysis (Figure 5) showed CuO to be present in the products of the combustion of all the samples. The set of reflections at 2θ = 32.68, 35.73, 38.89, 48.91, 53.70, 58.29, 61.71, 66.04, and 68.25 was consistent with ICSD Card No. 16025. In the G/Cu#CuO sample, the residue was as much as 49.3 wt%, indicating that polymeric matter (a phase susceptible to thermal decomposition) represented only half of the initial sample. 

At the end of the studies carried out in air atmosphere, it is necessary to comment on the effect that the physical form of the sample has on the results of thermal analysis. When carrying out comparative tests for samples of the M series that could be analyzed as grinded or granulated, no significant differences were found in the course of TG/DTG curves. In the next series of tests (under N_2_), the samples were in the same physical form as under air.

### 3.3. Thermal Analysis in Nitrogen 

**Figure 6:**
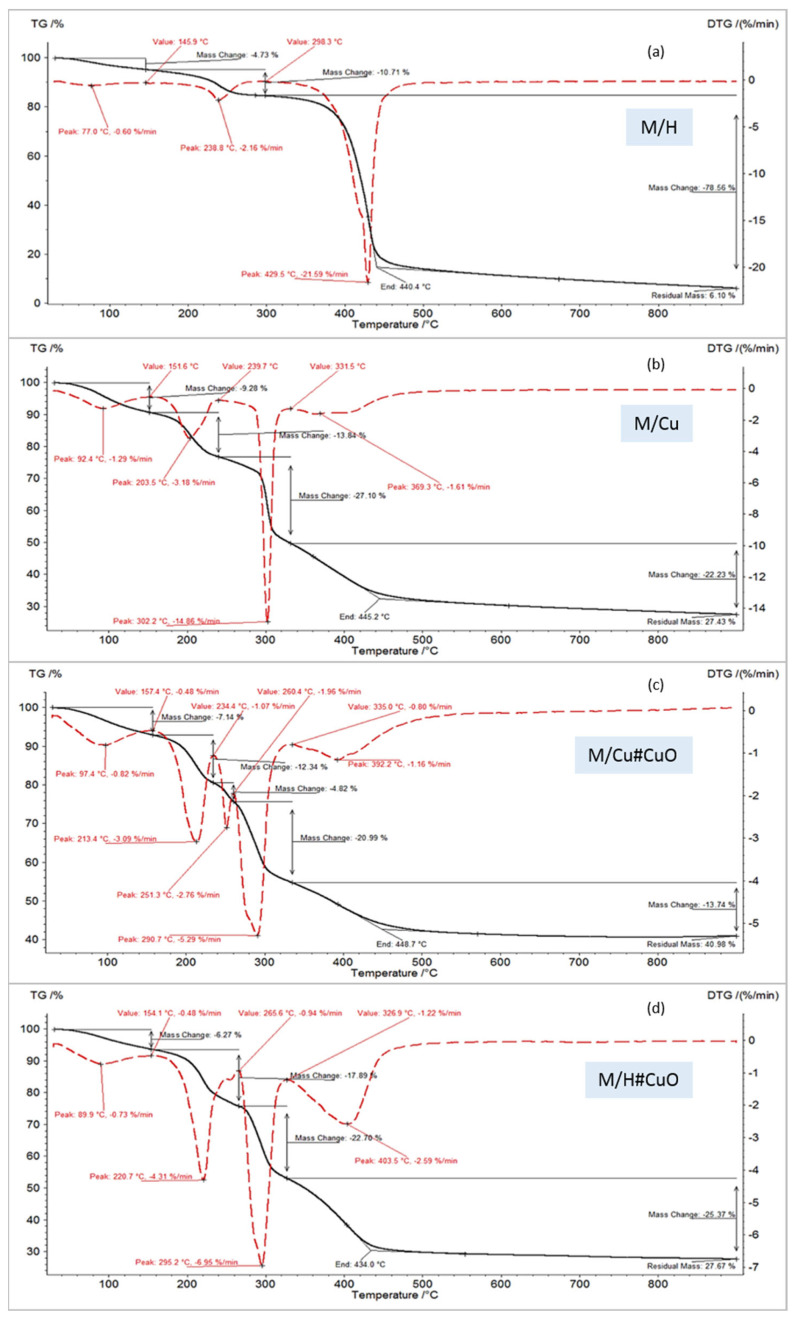
TG/DTG curves in N_2_ of (**a**) M/H, (**b**) M/Cu, (**c**) M/Cu#CuO, and (**d**) M/H#CuO.

**Figure 7:**
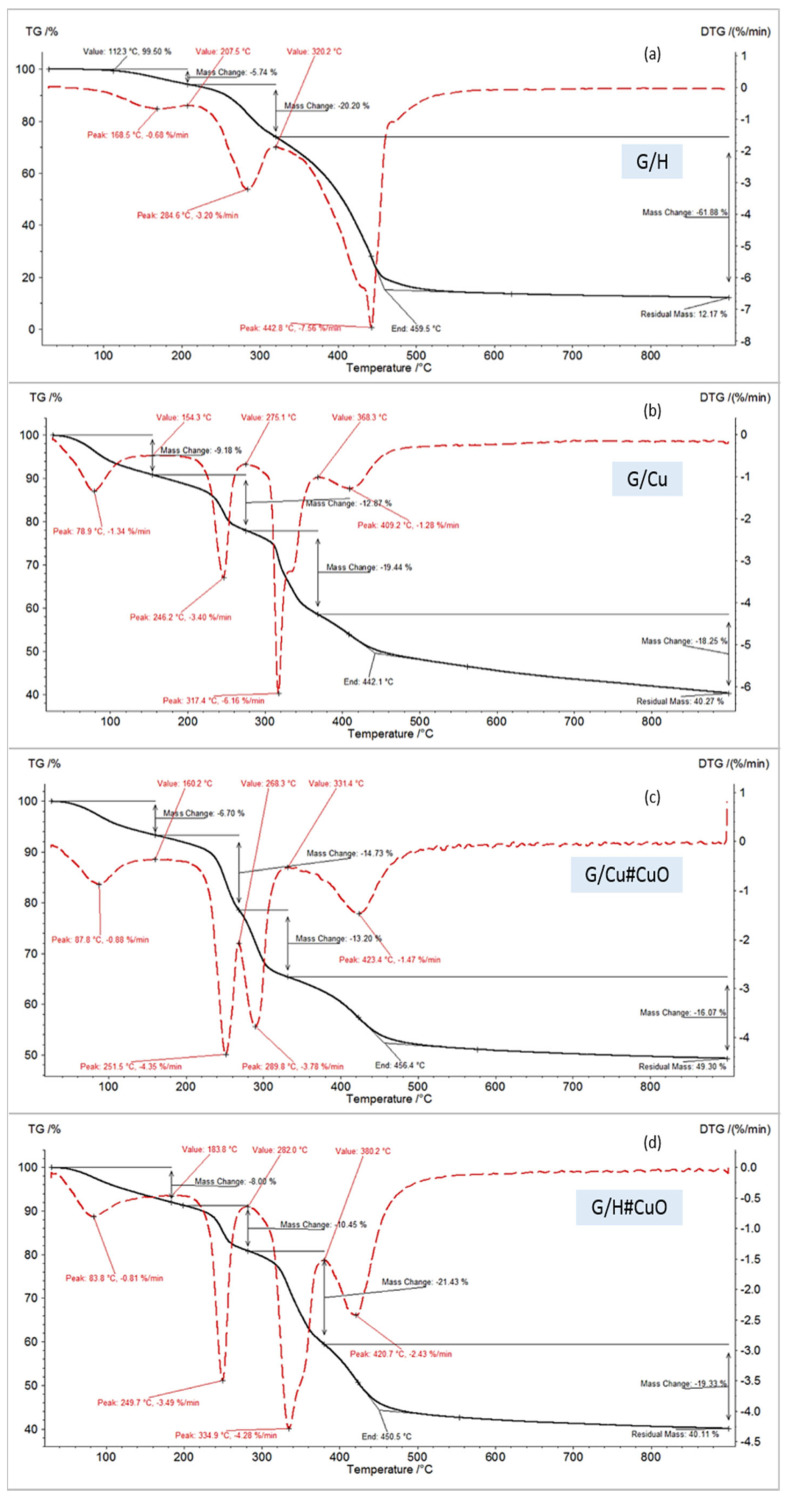
TG/DTG curves in N_2_ of (**a**) G/H, (**b**) G/Cu, (**c**) G/Cu#CuO, and (**d**) G/H#CuO.

Figure 6 and Figure 7 show TG/DTG curves of the same eight samples but recorded in N_2_ atmosphere. Up to 400 °C, both pure resins (M/H, G/H) decomposed just like in air (Figure 6a and Figure 7a vs. Figure 3a and Figure 4a). However, at about 400 °C, there was only one intensive transformation in N_2_ (in air there were two separate transformations), including decarboxylation and decomposition of the polymeric matrix (loss in mass 71.0 wt% for M/H and 62.0 wt% for G/H). For M/H and G/H, the end temperature in N_2_ was significantly lower than in air. The solid residue from M/H pyrolysis accounted for approximately 6.0 wt%. The solid residue from G/H pyrolysis (12.0 wt%) had an unusual form of foam/sponge, which “tried to get out” of the crucible (Figure 2, last column). As one can see in Figure 6b–d and Figure 7b–d, all HIXs in N_2_ decomposed in four stages (as in air); they decomposed much faster than the reference material (pure resin) and slightly slower (at a slightly higher temperature) than in air. 

**Figure 8:**
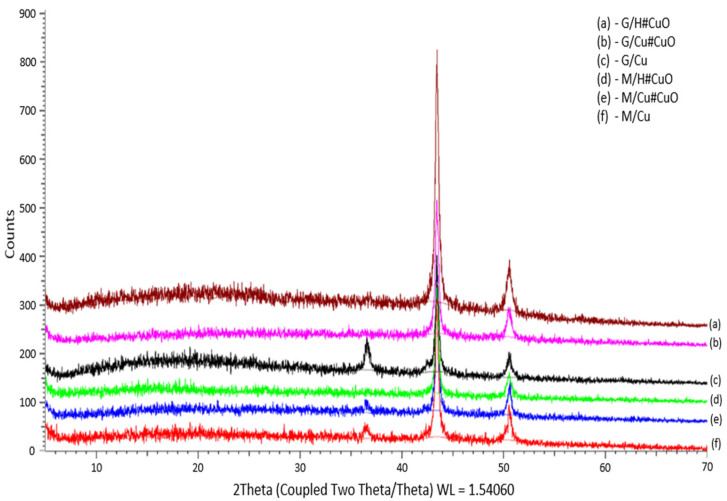
XRD patterns of residues in N_2_.

The interesting issues of the pyrolysis of six HIXs are connected with the composition and amount of the solid residue (pyrolysate). The pyrolysate has a much larger mass than the ash (the post-combustion residue) since, besides a mineral phase, it contains an organic phase (carbon char). More precisely, the pyrolysate contains carbon char (a mixture of various high molecular weight organic compounds constituting a product of the decomposition of the polymer’s matrix in air-free conditions) and inorganic matter (mainly deriving from the cupric deposit). Our previous study showed that during pyrolysis, the particles of copper compounds such CuO, Cu (OH)_2_, and Cu_2_O contained in the HIX received from anion exchangers had undergone reduction to Cu^0^ [40,41]. Although, in this case, the mass of cupric deposit in the skeleton was several times greater, the result was the same. Figure 8 shows six XRD patterns revealing that the inorganic load consisted of metallic copper (peaks at 2θ = 43.44 and 50.57, ICSD Card No. 43493). This is a valuable result as it means that, during pyrolysis, the whole of Cu (II) in CCE has been reduced to metallic copper (not to a mixture of copper compounds at different oxidation levels), for which a large amount of reducer (above 10.0 meq/g) was needed. In the photographs of pyrolysates (Figure 1 and Figure 2, last column), the characteristic color of metallic copper can be seen. 

The pyrolysates obtained during this study stand out in terms of mass and chemical composition. For the first time, we obtained pyrolysates with mass that represented as much as 50 wt% of the initial sample. For the first time, we obtained pyrolysates in which the inorganic phase (which was metallic copper) dominated. The high content of metallic copper in pyrolysate was due to the high Cu (II) content in the material subjected to pyrolysis and the process conditions for quantitative reduction. Comparing “solid residue” and “Cu content in respective starting sample” (Table 2 and Table 3), it can be estimated that HIX/M pyrolysates contained 70–80% wt% Cu and HIX/G pyrolysates 60–70% wt% Cu. As in the previous research [40,41,60], it turned out again that under N_2_, more pyrolysate formed from HIXs than in the case of the same transformation of pure resins (M, G). The research presented here shows that the polymeric matter from which the carboxylic cation exchanger is made (in addition to carbon and hydrogen atoms containing oxygen atoms) during pyrolysis does not give as much carbon char as in the case of a hydrocarbonaceous (oxygen-free) polystyrene matrix of anion exchange resin. However, there is no doubt that during pyrolysis of the polyacrylic/polymethacrylic matrix, the products of its decomposition (free radicals and hydrogen) participate in creation of an additional amount of carbon char due to the consumption of a certain amount of hydrogen to reduce Cu(II) to Cu^0^. The data show that as a result of pyrolysis, HIXs richest in copper (M/Cu#CuO and G/Cu#CuO) formed approximately 100% more carbon char than during pyrolysis of the respective cation exchangers with no copper deposit. Though both CCEs contained as much as 40.0 wt% oxygen in the macromolecule (the hydrocarbon chain accounted for less than half of the macromolecules), they have proven to be reagents capable of quantitatively reducing cupric deposits. 

## 4. Conclusions 

Carboxylic cation exchangers with very high ion exchange capacity can be used as host materials for receiving copper-rich hybrid ion exchangers. Using simple transformations in ambient conditions, macroreticular and gel-like HIXs with CuO particles with up to 195.0 and 233.0 mg Cu/g content were obtained. As carboxyl groups in CCEs are weakly acidic, using a dilute acetic acid solution HIXs containing both the CuO deposit and the functional groups in the H^+^ form can be obtained. HIXs enriched with copper, containing the CuO deposit and the functional groups in the Cu^2+^ form, could also be obtained (such materials contained up to 35.0 wt% Cu). All composites (regardless of the ionic form of functional groups) were extremely electrified (attached to paper, “jumped” from the dish). 

Samples of both starting materials (M/H and G/H) and also M/Cu, M/Cu#CuO, and M/H#CuO can be thermally analyzed in natural form (beads). G/Cu, G/Cu#CuO, and G/H#CuO samples were ground before measurement (otherwise they “pop out” from the measuring crucible).

The TG/DTG curves both CCEs and HIXs with the cupric deposit had a peculiar, characteristic course. Carboxyl groups attached to a skeleton with different structures (with polymer chains located close to each other or separated by macropores in the macromolecule) dehydrated/decarboxylated in different ways to intermolecular or intramolecular dehydration. 

Under air, the copper deposit significantly accelerated the decomposition of CCEs in such a way that subsequent changes occurred at a lower temperature, and the end temperature of ultimate polymeric matter decomposition was also lower (551.7 vs. 427.7 °C). For CCE in the Cu^2+^ form and for CCE containing dispersed CuO, the TG/DTG curves had a similar course. Materials with both Cu^2+^ and CuO contained the most copper and decomposed the fastest. For all analyses carried out under air, CuO was the solid residue. Its mass was up to 46.3%.

Under N_2_, all HIXs decomposed much faster than the reference material (pure resin) and slightly slower (at a slightly higher temperature) than in air. During pyrolysis, pyrolysates were formed with mass that represented as much as 50 wt% of the sample’s starting mass, consisting mainly of the inorganic phase (not organic as is usually the case), which was metallic copper (60–80% Cu). The creation of metallic copper was due to the reduced division into the cupric deposit of the products of polymer matrix thermal decomposition (free radicals and hydrogen). Simultaneously, free radicals participated in the creation of an additional amount of carbon char due to the consumption of a certain amount of hydrogen to reduce Cu(II) to Cu^0^. CCEs, despite their high oxygen content (about 40.0 wt%), have proven to be very effective reducers in the pyrolysis process.

## Figures and Tables

**Table 1:** Characterization of polymeric materials.

Properties	Amberlite IRC 50	Amberlite IRC 86
Appearance	Beige opaque spherical beads	Clear amber translucent spherical beads
Particle size, mesh	16–50	20–50
Matrix type	Methacrylic acid–DVB	Acrylic acid–DVB
Matrix structure	Macroporous (M)	Gel-like (G)
Functional groups	Carboxylic in H^+^ form (M/H, G/H)	
Total exchange capacity by dry weight, meq/g	10.5	10.7
Total reversible swelling,%	H^+^ → Na^+^, 100	H^+^ → Na^+^, 80

**Table 2:** Data of the transformations of carboxylic cation exchangers into HIXs with cupric deposit.

Stage	Substrate		Product		Mass Growth, %	Cu Content in Sample		Colour of the Product
	Code	Mass, g	Code	Mass, g		meq/g	mg/g	
**Macroreticular cation exchanger as polymeric support**								
A, B	M/H	9.36	M/Cu	13.54	+44.5	7.10	225.6	Light blue
C	M/Cu	10.01	M/Na#CuO	11.85	+18.2	5.07	161.0	Dark grey
D	M/Na#CuO	4.08	M/Cu#CuO	4.26	+4.4	10.30	327.3	Grey blue
E	M/Na#CuO	4.03	M/H#CuO	3.10	-23.1	6.15	195.4	Grey
**Gel-like cation exchanger as polymeric support**								
A, B	G/H	9.42	G/Cu	13.71	+45.5	7.21	228.9	Dark green
C	G/Cu	10.35	G/Na#CuO	12.52	+20.1	5.76	182.9	Black
D	G/Na#CuO	4.31	G/Cu#CuO	4.49	+4.17	11.16	354.4	Black
E	G/Na#CuO	4.17	G/H#CuO	3.22	−22.8	7.35	233.3	Black

**Table 3:** Thermal decomposition results.

Code	Water Mass Loss, %	Peak Temperature of Polymer Degradation, °C	End Temperature, °C	Residual Mass, %
decomposition in air				
M/H	4.02	237.8, 416.2, 487.2	503.8	0.89
M/Cu	9.37	221.9, 279.3, 362.6	412.4	26.65
M/Cu#CuO	7.27	216.6, 269.2 370.9	420.0	39.19
M/H#CuO	6.30	219.9, 281.7, 373.7	428.3	24.52
G/H	6.04	285.0, 427.1, 527.6	551.7	0
G/Cu	10.22	246.4, 313.9, 381.7	464.5	29.86
G/Cu#CuO	7.55	251.7, 276.4, 380.6	427.7	46.31
G/H#CuO	9.26	250.3, 318.5, 391.6	440.1	30.13
decomposition in N_2_				
M/H	4.73	238.8, 429.5	440.4	6.10
M/Cu	9.28	203.5, 302.2, 369.3	446.2	27.43
M/Cu#CuO	7.14	213.4, 251.3, 290.7, 392.2	448.7	40.98
M/H#CuO	6.27	220.7. 296.2, 403.5	434.0	27.67
G/H	5.74	284.6, 442.8	459.5	12.17
G/Cu	9.18	246.2, 317.4, 409.2	442.1	40.27
G/Cu#CuO	6.70	251.5, 289.8, 423.4	456.4	49.30
G/H#CuO	8.08	249.7, 334.9, 420.7	450.5	40.11

## Data Availability

The data are available from the corresponding author upon request.
